# Role of the Encapsulation in Bioavailability of Phenolic Compounds

**DOI:** 10.3390/antiox9100923

**Published:** 2020-09-26

**Authors:** Josipa Grgić, Gordana Šelo, Mirela Planinić, Marina Tišma, Ana Bucić-Kojić

**Affiliations:** Faculty of Food Technology Osijek, Josip Juraj Strossmayer University of Osijek, F. Kuhača 18, HR-31 000 Osijek, Croatia; jgrgic2@ptfos.hr (J.G.); gselo@ptfos.hr (G.Š.); mplanini@ptfos.hr (M.P.); mtisma@ptfos.hr (M.T.)

**Keywords:** bioaccessibility, simulated gastrointestinal digestion, target delivery, controlled release, encapsulation techniques, coating materials

## Abstract

Plant-derived phenolic compounds have multiple positive health effects for humans attributed to their antioxidative, anti-inflammatory, and antitumor properties, etc. These effects strongly depend on their bioavailability in the organism. Bioaccessibility, and consequently bioavailability of phenolic compounds significantly depend on the structure and form in which they are introduced into the organism, e.g., through a complex food matrix or as purified isolates. Furthermore, phenolic compounds interact with other macromolecules (proteins, lipids, dietary fibers, polysaccharides) in food or during digestion, which significantly influences their bioaccessibility in the organism, but due to the complexity of the mechanisms through which phenolic compounds act in the organism this area has still not been examined sufficiently. Simulated gastrointestinal digestion is one of the commonly used in vitro test for the assessment of phenolic compounds bioaccessibility. Encapsulation is a method that can positively affect bioaccessibility and bioavailability as it ensures the coating of the active component and its targeted delivery to a specific part of the digestive tract and controlled release. This comprehensive review aims to present the role of encapsulation in bioavailability of phenolic compounds as well as recent advances in coating materials used in encapsulation processes. The review is based on 258 recent literature references.

## 1. Introduction

Phenolic compounds are one of the largest groups of natural compounds and possess a wide range of biological activities (antioxidative, anti-inflammatory, anti-allergenic, antiviral, anticancer, antimicrobial, antimutagenic and cardioprotective, etc.) related to their structural diversity [1]. In the last 30 years, research has been mainly focused on: (a) determination of the phenolic compounds profile from various plant materials [2,3]; (b) determination of biological activity, especially antioxidant activity [4] and more recently antitumor activity [5]; (c) development and optimization of different extraction methods for phenolic compounds [1,6].

However, recent research is increasingly focused on shedding a light on mechanisms of how phenolic compounds are metabolized and become bioavailable in the human body [7].

The bioavailability, bioaccessibility, and bioactivity of phenolic compounds is currently the hot topic of scientific research since this area is quite unclear. These three terms are often confused, but they have different meanings. The bioavailability includes bioaccessibility and bioactivity (digestion, absorption, metabolism, distribution into the tissue, potential biotransformation, and physiologic response of the human body). The bioavailability of active substances originating from food can be defined as the rate and degree of absorption of the active matter through epithelial cells of the gastrointestinal tract (GIT). It has been proven that the metabolites of a compound can show completely different bioavailability compared to the initial food compound [8,9].

However, the actual bioavailability determination of certain active substances (e.g., phenolic compounds) in food products is complicated due to impracticality and ethical issues in research. Therefore, most research in this area is focused on bioaccessibility, which is defined as the content of the active compound that is released from food into the digestive tract and can potentially be absorbed or bioavailable [9,10]. Bioaccessibility is the key factor upon which bioactivity of phenolic compounds of different formulations (e.g., pharmaceuticals and functional food) depends on. Namely, the bioactivity of phenolic compounds determined via in vitro conditions can differ significantly from the bioactivity determined by in vivo conditions, which is attributed to the lower bioaccessibility of certain phenolic compounds in the digestive system. The reasons for lower bioaccessibility can be poor stability of compounds in aqueous solutions, poor stability in the GIT, and challenging absorption through membranes [9].

It is well known that phenolic compounds interact with other macromolecules during the digestive process, such as fibers, lipids, proteins, and food polysaccharides, which affect their bioaccessibility. Bioaccessibility is most commonly assessed through the processes of simulated digestion in the GIT in vitro [9]. The bioaccessibility of phenolic compounds depends on the chemical structure of the compound, the degree of glycosylation, acylation, conjugation with other phenolic compounds, degree of polymerization, and solubility. It has been recognized that bioaccessibility of high-molecular-weight phenolic compounds (e.g., hydrolyzable and condensed tannins), complex flavonoid conjugated with sugars and acetylated with hydroxycinnamic acids is lower compared to aglycones (units without sugar) and low-molecular-weight polyphenols [10].

Furthermore, various food processing can change the nutritional value of food and the bioavailability of biologically active ingredients (e.g., phenolic compounds), in a positive or negative direction. By applying certain procedures during processing, these changes are aimed at preserving or increasing the nutritional and biologically active properties, which is especially desirable during the production of functional foods and food supplements.

Encapsulation is a process where the active matter or their mixture is coated with a polymer that protects it from negative external influences and that allows the controlled release of active matter in a certain ambiance [11]. The controlled release of active matter is an important property of phenolic compounds bioavailability in the organism. Additionally, encapsulation can cover unwanted odors and tastes, and apart from improving technological and health benefits, it can contribute to better sensory properties of encapsulated products [12]. Encapsulation efficiency can be influenced by many factors such as encapsulation technique, properties of coating materials and active compounds, interactions between compounds, etc. [13].

The problems encountered by the researchers are emphasized and it was suggested in which direction further research should progress. This comprehensive review aims to present the role of encapsulation in bioavailability of phenolic compounds as well as recent advances in coating materials used in encapsulation processes. The review is based on very recent bibliographic references where most of them are within the period 2009–2020, and a final database of 258 references was used.

## 2. Phenolic Compounds

Phenolic compounds are the most widely distributed secondary metabolites produced via the shikimic acid pathway in plants during their growth or as a response to environmental stress conditions (e.g., microbial infections, injures, or UV radiation). Their content in the plant depends on the plant variety, growing conditions, ripening, harvest, as well as on the processing and storage conditions [12,14]. The term “polyphenols” is often used in the literature as a synonym for phenolic compounds, which is not entirely accurate and can be confusing while this term excludes phenolic compounds with a single aromatic ring. There are many classifications of phenolic compounds owing to their high structural diversity, but common to all groups is that they have at least one aromatic ring with one or more hydroxyl groups. The main classes are simple molecules with one aromatic ring (phenolic acids) and polyphenols with two or more aromatic rings (flavonoids, lignans, and stilbenes) [15,16]. Other plant phenolics comprise coumarins, hydrolyzable, condensed tannins and lignins, etc. Phenolic compounds can exist in plants in different forms, in a non-glycosylated form (aglycone) or as glycosides, linked with organic acids or with other complex molecules such as tannins [17].

Dietary phenolic compounds are daily consumed from various sources such as vegetables (onion, broccoli, cabbage), fruits (citrus, grapes, berries), and drinks (coffee, tea, wine). They are responsible for different properties of food such as flavor, odor, color, and astringency [14,16]. Since the 1990s, phenolic compounds raised a lot of interest because of their benefits for human health. Many in vivo and in vitro assays have been developed for their health benefits assessment [18]. Except from well-known antioxidant activity [19,20], phenolic compounds also have, among others, cardio-protective [21], anti-cancer [22], anti-diabetic [23,24], and neuroprotective [25] effects. The most recognized representatives of bioactive phenolic compounds in literature are resveratrol, epigallocatechin gallate (EGCG) and curcumin. These compounds have been investigated mostly due to their positive contribution to human health, particularly due to the risk reduction of various chronic diseases [26,27,28].

### 2.1. Phenolic Acids

**Figure 1:**
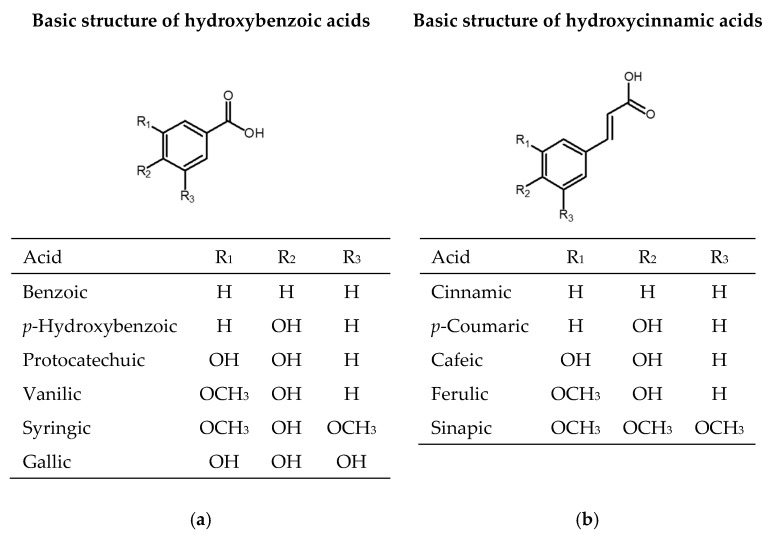
Chemical structures of phenolic acids: (**a**) derivates of hydroxybenzoic acid; (**b**) derivates of hydroxycinnamic acid.

One-third of the dietary phenolic compounds belong to phenolic acids, which are present in fruits, vegetables, and cereals. Phenolic acids contain one aromatic ring and at least one organic carboxylic acid group. They can be divided into benzoic acid derivatives with C6-C1 structure (gallic, *p*-hydroxybenzoic, protocatechuic, vanillic, syringic, benzoic acids) and cinnamic acid derivates with C6-C3 structure (cinnamic, *p*-coumaric, caffeic, ferulic, sinapic acids) (Figure 1). They appear in plants as esters or glycosides conjugated with other natural compounds or to a lesser extent in the free acid form [29].

### 2.2. Flavonoids

**Figure 2:**
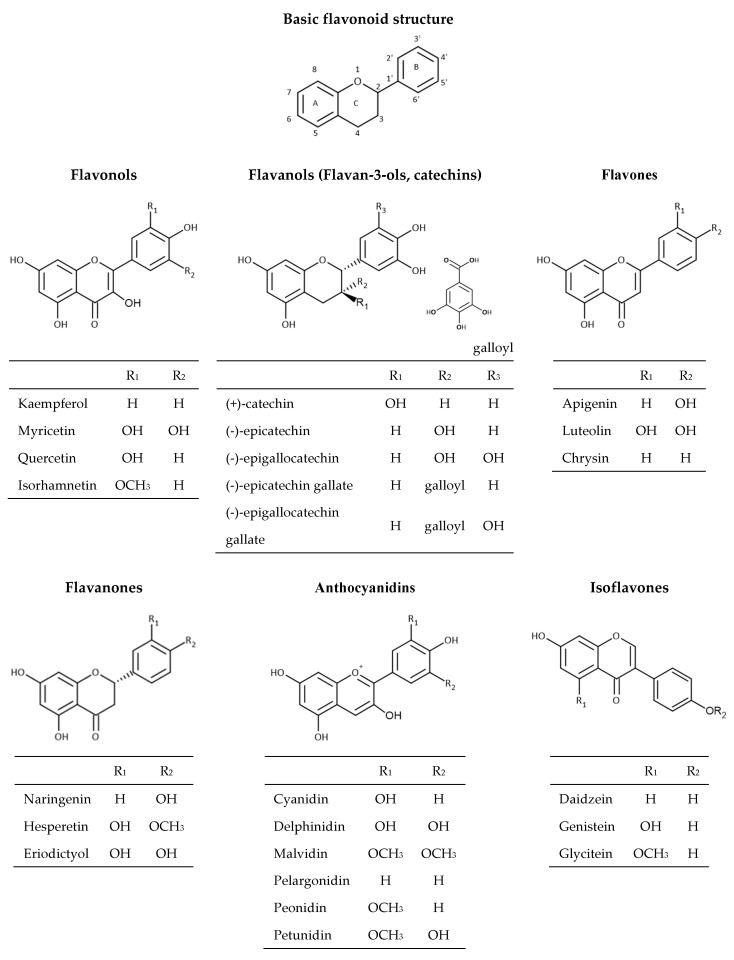
Chemical structures of flavonoids.

Flavonoids are the most abundant group of polyphenolic compounds in the plants. Flavonoid structures are based on a 15 carbon skeleton (C6-C3-C6) consisting of two benzene rings A and B linked via oxygencontaining heterocyclic ring C (Figure 2).

**Figure 3:**
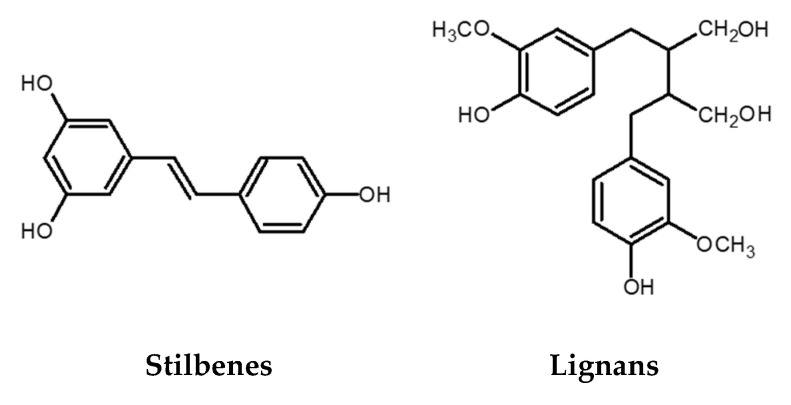
Chemical structures of stilbenes and lignans.

The position of different substituents (hydroxyl, methoxy, and glycosidic groups) on rings A, B, and C, the binding position of rings B and C, and the unsaturation degree and degree of oxidation in the structure of ring C is responsible for their structural diversity and multiple biological activities [30]. Dietary flavonoids can be divided into six groups (Figure 3) based on the oxidation of the heterocyclic ring, such as flavones (apigenin, luteolin, etc.), flavanones (naringenin, hesperetin, eriodictyol), flavonols (quercetin, kaempferol, myricetin, etc.), flavanols (catechin, epicatechin, epicatechin gallate, etc.), anthocyanins (cyanidin, delphinidin, malvidin, peonidin, etc.), and isoflavones (genistein, daidzein, etc.) [16,31]. Flavonoids in plants are mainly found in the form of 3-*O*-glycosides or polymers (tannins). The most common glycoside units are glucose, glucoramnose, galactose, arabinose, and rhamnose. Polymer compounds can originate from the plants themselves (condensed tannins or proanthocyanidins and hydrolyzable tannins) or can be formed during food processing (derived tannins) [32,33].

### 2.3. Stilbenes

The stilbene structure is based on the C6-C2-C6 backbone, which consists of two aromatic rings linked by an ethylene bridge (Figure 3). One of the extensively studied stilbenes is resveratrol owing to its wide range of biological activities including adipocytes lipolysis, anti-inflammatory activity, anticancer activity, protection against cardiovascular and neurodegenerative pathologies modulation of cell proliferation and angiogenesis, among others. Grape and its processed products (e.g., wine) are rich sources of resveratrol [14,34]. Besides resveratrol itself, its oligomers also possess a wide range of biological activities (antibacterial, anticancer, antioxidant activity, anti-HIV, etc.) [35,36,37]. An example of an oligomer, more specifically a resveratrol dimer, is *ε*-viniferin. Various studies have shown that this polyphenol plays an essential role in the inhibition and progression of cancer [38]. In addition to *ε*-viniferin, another resveratrol dimer, pallidol, has also been shown to exhibit stronger antioxidant activity than resveratrol alone [37,39,40].

### 2.4. Lignans

Lignans are phenolic dimers possessing a 2,3-dibenzylbutane structure (C6-C3-C3-C6 structure) (Figure 3). They participate in the formation of the building blocks for the formation of lignin, which is responsible for the structural rigidity of the plant cell wall. Seeds (linseeds, flaxseeds), followed by nuts, grains, vegetables, tea, coffee, and wine are a rich source of lignans. They have anticancer, antioxidant, antihypertensive, antiviral, estrogenic, and insecticidal properties [41].

## 3. Bioavailability, Bioaccessibility, and Bioactivity of Phenolic Compounds

**Figure 4:**
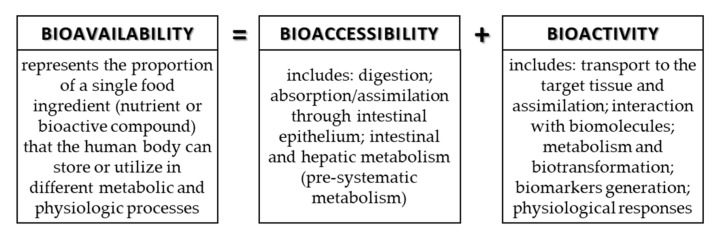
Connection of terms: bioavailability, bioaccessibility, and bioactivity.

The food contains many components (nutrients and bioactive compounds) that can be absorbed during digestion, but to be available for absorption/assimilation, these components, or their fractions, first have to be released from the food matrix [9,42]. The release of components and/or their fractions to make them available for absorption during digestion in the GIT is the definition of bioaccessibility. Bioaccessibility is the first step to make an ingredient bioavailable. In addition to bioaccessibility, bioavailability includes the usage of the ingredients and its bioactivity, which includes the outcome that the absorbed component has (Figure 4). More precisely, bioavailability includes GI digestion, absorption, bloodstream, and tissue distribution, and, finally, bioactivity [8,9].

Due to the diverse structure of phenolic compounds and their different bioactivity, the positive effects of phenolic compounds cannot be fully exploited. Furthermore, foods and their processing, the complexity of the food matrix, and possible synergistic interaction of phenolic compounds with other components cause reduced bioavailability [43,44].

Numerous in vitro tests are used to determine the biological activity of phenolic compounds from plants [45,46,47]. Although for some phenolic compound in vitro tests can give favorable results in biological activity (e.g., antioxidant activity), in vivo tests can often give completely different results. For the sake of in vitro tests, high concentrations of phenolic compounds (µmol/L or mmol/L) are usually used to “force” positive results, while the results obtained with in vivo tests are expressed in nmol/L if the daily intake of the studied compound is observed [48]. Additionally, different results in bioavailability are obtained due to the fact that phenolic compounds are unstable under conditions found in the GI tract, insufficiently soluble and most of them cannot pass through the intestinal membrane [49,50].

### 3.1. Factors That Affect Bioavailability of Dietary Phenolic Compounds

Regarding the complex structure of the produce, phenolic compounds are not evenly distributed and many factors affect their bioavailability [51]. Dietary phenolic compounds are usually found in larger quantities in the outer layers of fruit and vegetables, and therefore, often remain unconsumed. There are factors that affect the content of phenolic compounds in food, such as growing conditions, harvest conditions, food processing, and food-related factors. Host-related factors (age and gender, physiological conditions, colonic microflora, enzyme activity, etc.) play a great role in bioavailability [52]. Phenolic compounds interaction with other compounds during ingestion is an important factor of bioavailability, too.

The solubility is a physicochemical property of phenolic compounds that significantly affect their bioavailability. Plant phenols are distributed differently in plant tissue. Accordingly, soluble phenolics are found in cell vacuole, while insoluble ones are within cell walls [51]. A common confusion may be the belief that more soluble substances also have higher bioavailability, but the permeability of intestinal membranes plays a major role. According to this, phenolic compounds can be classified into three groups, where curcumin is a representative of a group of phenolics that have both low solubility and membrane permeability. Resveratrol also has low solubility, but, compared to curcumin, it is characterized by high membrane permeability. The last group belongs to phenolic compounds with low membrane permeability but high solubility such as EGCG [53,54].

The food matrix has a big impact on bioaccessibility and therefore bioavailability of phenolic compounds. In vivo tests, where sucrose and ascorbic acid were used, have shown that the bioavailability of two catechins, epigallocatechin (EGC) and EGCG, can be improved. In contact with the GIT juices, sucrose can cause an increase of viscosity, which improves catechins uptake by lumen. Ascorbic acid affects the stability of catechins against oxidative degradation and a reduction in the efflux of absorbed catechins back into the lumen [55].

Furthermore, it was shown that by a simultaneous intake of milk and blueberries, milk reduces the recovery of total phenolics and anthocyanins [46]. Binding skim milk protein and green tea catechins lead to a decrease in catechins recovery. However, at the same time, research conducted on Caco-2 cells showed an increase in catechins intake by the addition of skim milk [56]. Fats, along with proteins, may positively affect the bioavailability of phenolic compounds due to possible micellarization in the small intestine [42].

The bioavailability differs for each phenolic compound, but the chemical structure is the one that determines the rate and extent of phenolic compound absorption, not the concentration. Most of the phenolic compounds are found as esters, polymers, or glycosides in food, and in that form, they cannot be absorbed. Additionally, after the ingestion of produce rich in phenolic compounds, gut microbiota, enzyme modifications, and bloodstream transport affect their bioavailability. The low absorption rate can be explained as a consequence of phenols’ poor water solubility and stability in different conditions. Compounds with higher molecular weight, such as proanthocyanidins, which contain catechin and epicatechin units, are not well dissolved in water and have a lower absorption rate [49,57].

### 3.2. Digestion in GIT

The digestive system consists of a multitude of organs that play a role in the breakdown and absorption of ingested food. During the process of digestion, food is cleaved, not only in the mouth, but also by contractions of the GIT. Cleavage allows the release of nutrients and bioactive components that become available for absorption. Additionally, the juices found in the stomach and small intestine promote the release of enzymes that catalyze the breakdown of carbohydrates, proteins, fats, and smaller components such as phenolic compounds, carotenoids, etc. Intestinal movements are involved not only in the breakdown but also in the absorption and transport of unabsorbed components.

It is important to mention that the colon contains a large number of microorganisms, 10^12^ microorganisms per gram of gut content. Gut and its microbiota for long have been thought to be an insignificant part of the human body, but more recent research suggests just the opposite. Today, it is known that the gut microbiota has many functions that the human host cannot achieve on its own, and this is precisely the reason why the human gut microbiota is considered a “superorganism” [58].

Further sections briefly describe the metabolism of phenolic compounds through the different stages of digestion.

#### 3.2.1. Oral Cavity

Digestion of phenolic compounds begins in the oral cavity where phenolics are released from the food matrix. The released phenolic compounds come in contact with saliva. Saliva affects some phenolic compounds, such as flavanol gallate esters, which are affected by degalloylation, while other phenolic compounds, such as catechins, remain unchanged [59]. The pH prevailing in the mouth depends on the food and drink ingested, but it is usually in the range from 6 to 7. If the phenolic compounds are bound to a sugar unit, it is the bound sugar that will affect the absorption. Phenolics containing sugar moiety (glycosylated form) can be deglycosylated in the mouth due to residual bacteria found in oral epithelial cells [59]. Glucose from glucosinolate precursors found in cruciferous vegetables undergoes enzymatic hydrolysis by myrosinase. These precursors are hydrolyzed to isothiocyanate [59]. Amylase, the most dominant enzyme in saliva, has no great impact on phenolic compounds [59,60,61].

Acidic proteins, rich in the amino acid proline, found in the oral cavity, can bind to phenolic compounds with hydrogen bonds or hydrophobic interactions. Examples are proanthocyanidins and flavanols, which form a polyphenol-protein complex whose feature is solubility. Binding of polyphenols changes the secondary and tertiary structure of the proteins themselves [62]. Despite the formation of soluble protein polyphenol complexes, the availability of most polyphenolic compounds does not change much [60]. Tannins are also renowned to bind with saliva proteins, and it is the formation of tannin-protein complex that affects their bioavailability [63,64]. Upon chewing, the size of the particles is reduced, which allows better enzyme access during further digestion stages.

#### 3.2.2. Upper Part of the GIT

As digestion progresses, there is a further reduction in food particle size, which helps the release of phenolic compounds. Most phenolic compounds are released from food particles during gastric digestion, and some can be absorbed in the stomach in their free form, such as phenolic acids. Except for the absorption of some phenolic compounds in the stomach, hydrolysis and deconjugation also take place [59,61]. After stomach digestion, non-absorbed phenolic compounds pass to the small intestine and pH changes from acidic (pH 2–4) to neutral (pH 7). Acidic conditions present at the beginning of small intestine digestion may favor the presence of the phenolic compounds [65].

It is estimated that only 5–10% of phenolic compounds, ingested through food, are absorbed in the small intestine, while others reach the large intestine [66,67,68]. Since phenolic compounds are mostly in polar form as glycosides, their absorption in the small intestine is unlikely, but those that undergo hydrolysis and become aglycones can pass to the liver through the portal vein [42,48,61]. As the pH rises, enzymes secreted by the pancreas and bile activate and contribute to the absorption of phytochemicals especially bile salts and lipase. These enzymes abet the digestion of apolar components, such as phytochemicals or lipids, and in this way water-soluble micelles can be formed [61,65,69,70,71]. Aglycones of isoflavonoids and flavonoids can also be micellarized like this and that causes low bioaccessibility and therefore bioavailability [72]. Upon an increase in pH values, anthocyanins first lose their color and are then subjected to further decomposition by the opening of the C ring [61]. As the exact mechanism of anthocyanin absorption is not fully clear yet, two courses of action have been proposed. One is hydrolysis in the intestinal lumen to form aglycones. The resulting aglycones are then absorbed into the enterocytes by passive diffusion. Another way involves a sodium-dependent glucose transporter that helps the transport of anthocyanins across the brush bored membrane and hydrolysis by cytosolic β-glucosidase prior to transport into the blood [73].

Two enzymes found in the small intestine, lactase-phlorizin hydrolase (LPH) and cytosolic β-glucosidase (CBG), hydrolyze glycosylated flavonoids into aglycones [61,74]. LPH shows selectivity for flavonoid-*O*-β-d-glucosides. The resulting aglycones, by passive diffusion, make the way to epithelial cells due to increased lipophilicity and contiguity to the cell membrane [18].

Research conducted with Caco-2 cells and in cell-free buffers and media, simulating pH conditions of the small intestine, has shown that used cyaniding-3-*O*-glucoside was degraded. Decomposition of this anthocyanin, under given conditions, caused the formation of phloroglucinol aldehyde and protocatechuic acid [59].

There are a few exceptions, for example, flavonoids that are bound to the rhamnose unit, which are carried into epithelial cells by passive diffusion. They reach the colon and are hydrolyzed by *α*-rhamnosidase, which is secreted by the colon microbiota. In addition to passive diffusion, aglycones can also reach enterocytes by active or facilitated transport. Passive diffusion transmission is characteristic of low molecular weight phenolic compounds [75] such as phenolic acids, epicatechin, procyanidin B2, catechin, and others, as suggested in studies with Caco-2 cell trials. With the help of sodium-glucose-linked transporter 1 (SGLT1), some phenolic compounds in the form of glycosides can also be transported. Facilitated transport of phenolic compounds is carried out by monocarboxylic acid transporters (MCTs). In order for MCTs to recognize them as substrates, the phenolic compounds must contain a monoanionic carboxylic acid group and a non-polar side chain or aromatic hydrophobic unit. According to the research where models with Caco-2 cells have been used, it has been shown that the transport of caffeic and ferulic acid via these transporters is possible [61].

There are few possibilities for high molecular weight polymers, such as proanthocyanidins, to be absorbed in the small intestine. Hydroxycinnamic acids, another large group of phenolic compounds, are often esterified. As there are no esterases in the tissues, the main place where their metabolism takes place and where the ester bonds that bind them to organic acids, sugars, and lipids can be broken, is the colon [61,74]. Some hydroxycinnamic acids, however, can be absorbed in the small intestine. Esters of phenolic acids have low bioavailability in comparison to the bioavailability of their free form, which implies that a greater part of esters would be sunder in the colon [61].

After absorption of aglycon or derivatives in the small intestine or colon, they undergo further metabolism at the enterocyte level. Phenolics reach the bloodstream either in free form or bound to other compounds such as proteins [61]. The degree of polymerization has been shown to have a significant effect on cellular uptake. An intake of about 45% epicatechin, which is a monomer, was found, while the absorption of procyanidin dimers is much lower [76,77]. Polymeric procyanidins and tannins are thought to be completely inaccessible for absorption [76,77,78]. It is noteworthy that in animal studies, tannins and polymeric procyanidins were not observed in urine after oral administration [78].

Further metabolism includes methylation, glucuronidation, and sulfation. Uridine diphosphate glucuronosyltransferases (UGT) are enzymes responsible for polyphenol glucuronidation in the small intestine. It is proposed that isoforms of UGT1A8 and UGT1A10 play important role in flavonoids glucuronidation [59]. Other important enzymes are catechol *O*-methyltransferases (COMT), which catalyze methylation of phenolic compounds that have catechol residue. Therefore, by the action of these enzymes, high concentrations of *O*-methylated flavanol glucuronides and other *O*-methylated forms are observed [59,79]. Via the portal vein, the products of these changes reach the liver, where metabolism continues. The products are conjugated and again transported into the bloodstream and excreted in the urine. The main change that phenolic compounds undergo in the liver is sulfation [60]. The enzymes that conduct sulfation are sulfotransferases (SULT) especially SULTA1 and SULTA3. An example of their catalytic action is the sulfation of B-ring of (-)-epicatechin [60]. Some conjugate products are excreted in the form of bile and returned by enteropathic recirculation to the intestine via ATP-binding cassette transporters. Monocarboxylate transporters and multidrug resistance proteins may also participate in transport to the vascular side as demonstrated by the Caco-2 cell assay [80].

The remaining unconjugated compounds are regenerated with the help of microbial enzymes from the intestines before re-absorption. The remaining unabsorbed components are removed in the feces. The efficiency of conjugation reactions is high and free aglycones present in plasma are either in small concentrations or not present at all [68,74]. Catechins aglycones from tea are found in a large proportion in plasma, which makes them an exception. This is because they are not in the glycosylated form in food and do not undergo changes in the small intestine.

#### 3.2.3. Colon

The proportion of absorbed phenolic compounds in the upper GIT is relatively small compared to intake [59,66,67,68]. Low absorption of phenolic compounds in the small intestine indicates that even those phenolic compounds that are conjugated and absorbed in enterocytes or liver will reach the large intestine [59,81]. The large intestine has various microorganisms [59] and the role of phenolic compounds in the colon and gut microbiota is reciprocal [82]. As the microbiota changes the chemical structure of phenolic compounds and participates in the formation of metabolites, thus changing their bioavailability, phenolic compounds are able to change the microbiota of the colon [81]. Enzymes produced by bacteria in the colon trigger various reactions to form new metabolites [18,61,68]. While microbiological fermentation negatively affects the bioavailability of phenolic compounds, newly formed metabolites are often more bioactive than their predecessors [67]. One example is dihydroresveratrol, derived from resveratrol, which has higher bioavailability of resveratrol itself [83].

Flavonols degradation rate in the colon depends on previous glycosylation, but also the position of hydroxyl groups. The type of glycosidic bond plays a major role in flavanol metabolism, where the C-glycosidic bond breaks down much more slowly than the *O*-glycosidic bond. This is of great importance given that those compounds that are more slowly degraded also have a higher bioavailability [74]. After being metabolized to aglycones, simpler phenolic compounds are formed from flavonols under the influence of gut microbiota. After flavonoid C-ring degradation, at the remaining A- and B-ring, dihydroxylation occurs. In this way, phenolic compounds are formed and absorbed in the large intestine. The colon microbiota affects flavanones to a lesser extent compared to flavonols and flavan 3-ols, making them more bioavailable. Flavanones are similarly degraded as flavonols, the only difference being in the C-ring cleavage [74].

Flavan-3-ols is a group of flavonoids consisted of complex compounds such as catechin, epicatechin, and EGC. Their bioavailability is affected by the degree of polymerization and galloylation [67,74]. The lack of a carbonyl group at the C4 site, such as that presented in flavonols and flavanones, may be the reason why the gut microbiota does not cause major changes to flavan-3-ols [74]. After gallate esters are metabolized, the C-ring of aglycone opens. In this reaction, diphenylpropan 2-diol is produced, which is later metabolized to 5-(3′,4′-dihydroxyphenyl)-*γ*-valerolactone, and then, by opening the lactone ring 5-(3,4-dihydroxyphenyl), valeric acid is formed. Further changes produce OH-phenylpropionic and hydroxy-benzoic acids [67,74].

Unlike flavonoids, anthocyanins are not subject to large changes in the colon, and therefore, their bioavailability is not as great. A small amount of anthocyanins is absorbed in the small intestine and larger amounts reach the large intestine, where they are deglycosylated [74]. Over the years, anthocyanin degradation products such as vanillic acid have been successfully observed [74,84]. Incubation of malvidin-3-glucoside with fecal bacteria has been shown to produce phenolic acids, specifically *p*-coumaric, gallic, and syringic acid [85]. Interestingly, anthocyanins, along with their metabolites, can positively affect the colon microbiota [86,87].

Most isoflavones are present in food in the form of glucosides and as such cannot be absorbed at the level of enterocytes because of their high polarity and high molecular weight. In order to be bioavailable, they must be transformed into aglycone form by β-glucosidases, and these aglycones go into the peripheral circulation [74].

Flavones in the form of glycosides are hydrolyzed in the small intestine and unabsorbed aglycones are metabolized in the large intestine. The colon microbiota allows C-ring cleavage to form compounds such as 4-hydroxycinnamic acid and various forms of propionic acid. These compounds, after absorption, are excreted in the urine [88].

Gallotannins and ellagitannins are representatives of the polyphenol group hydrolyzable tannins. The difference in the metabolism of these compounds is that during their hydrolysis in the colon, gallotannins release gallic acid and glucose, and ellagitannins produce ellagic acid during lactonization. The resulting ellagic acid is further metabolized to form urolithin A and B [89]. The amount of these compounds excreted in the urine depends on the colon microbiota [90,91]. The colon microflora does not affect the degradation of condensed tannins [61].

## 4. Encapsulation Techniques

Encapsulation is the process where small solid, gas, or liquid active agents (core) is being packed within wall material called shell [92]. Encapsulation products are called capsules or beads and they can vary in size and properties. If the capsules are smaller than 1 µm, they are called nanocapsules, while those with a size of 3–800 µm are microcapsules [18,93]. In the last few years encapsulation has raised a lot of interest. It can be used as a way to preserve bioactive compounds against degradation caused by environmental conditions (e.g., heat, light, air, and moisture) enhancing desirable food properties, or on the other hand, masking of unpleasant odor or flavor [92,94]. It can decrease the evaporation of the entrapped active agents, prevent the incompatibility between components of entrapped mixture, and modify the physical properties of original substances. Encapsulation is also used to enhance the bioavailability of bioactive compounds, such as drugs and phenolic compounds, in a way that using various wall materials enable target delivery and controlled release (delayed or long-acting release) [95]. Numerous encapsulation techniques suitable for food ingredients have been developed. When choosing the encapsulation technique, several things need to be considered, such as the material to be encapsulated (core) and the wall material that can be used. Then, the desired properties of the capsules are considered, such as morphology and their size. Optimizing these factors can significantly increase the efficiency of encapsulation. The following part briefly describes the most commonly used encapsulation techniques.

### 4.1. Encapsulation by Spray-Drying

**Figure 5:**
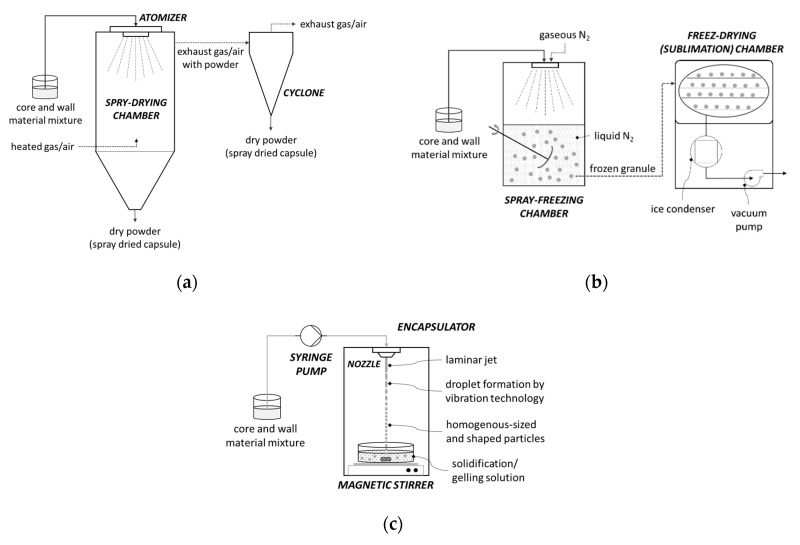
Scheme of some encapsulation procedure: (**a**) encapsulation by countercurrent spray draying, (**b**) encapsulation by freeze-drying (lyofilization), (**c**) encapsulation by extrusion with vibration technology for producing uniform microcapsules.

This technique is the most commonly used encapsulation technique due to its many advantages such as low cost, good quality of final product, and easy scale-up [90,93]. Core and wall material are prepared by mixing and then their mixture is passed through a nozzle or spinning wheel of spray dryer into a hot chamber where the mixture is atomized (Figure 5a). In contact with hot air, the solvent evaporates and the wall material solidifies around the core material. The resulting capsules are in the form of powder and they fall to the bottom of the device [96,97]. Spray drying is a convenient encapsulation technique due to the ability to encapsulate many components. Minerals and vitamins, flavors and colors, oils, and fats are just some of them. They provide protection to encapsulated material and often extend its shelf life. The disadvantage of this technique is the limited use of wall materials [98]. During the preparation, a mixture of wall and core material is made, and therefore, the wall material must be soluble in water [96].

### 4.2. Encapsulation by Freeze-Drying (Lyophilization)

With this encapsulation method, the material is frozen to form ice crystals, which are further sublimated in the vacuum chambers (Figure 5b). The remaining water is reduced by desorption, also known as secondary drying. Sublimation of ice crystals creates porous structures that later provide good rehydration properties of the powdered product [96]. It is a simple technique, most suitable for the encapsulation of aromas and other volatile compounds and for dehydrating materials sensitive to high temperatures that are unstable in aqueous solutions. The main disadvantages of freeze-drying are the long period of dehydration and the high economic and energy costs compared to spray drying [97,98,99,100,101].

### 4.3. Encapsulation by Extrusion

This is a process (Figure 5c) where a polymer solution containing the active component is passed through a nozzle into a gelling solution [102]. When the extrusion is used in the laboratory, syringes are most commonly used as nozzles. For this technique, sodium alginate is mostly used as wall material and capsules are formed in a solution of calcium chloride solution [103]. The size of the formed capsules varies depending on the diameter of the syringe used, but also on the flow rate at which the mixture of polymer and the active component is released [102]. There are many advantages of extrusion: it is easy to carry out on a laboratory scale, the resulting capsules have a long shelf life, and, the biggest advantage, it has the ability to encapsulate a wide range of components regardless of whether they are hydrophilic or hydrophobic. This method produces capsules that are large and porous which cause quick diffusion of encapsulated material, in addition, the materials used as wall materials are limited. Another disadvantage is that the scale-up of this method is expensive but also demanding to perform [104].

### 4.4. Encapsulation by Emulsification

An emulsion involves two immiscible liquids such as water and oil (e.g., water-in-oil or oil-in-water). One of two liquids is dispersed into the other as droplets [96]. This kind of encapsulation requires an emulsifier for emulsion stabilization. Encapsulated bioactive compounds after emulsification can be in liquid or powder forms if it is subjected to drying afterward [96]. This encapsulation method is suitable for oil-soluble compounds such as carotenoids, plant sterols, and dietary fats. The emulsification technique can be divided into nanoemulsions and double emulsions [96,104].

### 4.5. Encapsulation by Coacervation

The principle of the coacervation encapsulation technique is the separation of polyelectrolyte phases from the solution and the formation of coacervate around the core material. Polyelectrolyte can be stand-alone or in the form of a mixture [93]. The number of used polymers determines the coacervation type, meaning that when using only one polymer type, coacervation is simple, while complex coacervation includes using polymer mixture as wall material. To enhance firmness and stability of wall material, cross-linking agents, chemical or enzymatic, can be used [93,96,104]. The electrostatic interactions of the cross-linking agent help the formation of coacervates. The agents must be strong enough to induce electrostatic interactions, but at the same time, the interaction must not be too large to prevent precipitation [100]. Coacervation is a promising technique that allows high efficiency of core material encapsulation and controlled release, but the disadvantage is that resulting capsules are not stable in aqueous solutions and tend to agglomerate. Furthermore, the technique and process itself is expensive and pH-sensitive [96,103].

### 4.6. Encapsulation by Molecular Inclusion

This encapsulation technique is also known as host-guest complexation and it occurs at a molecular level. The principle of this method is that apolar guest molecules can be trapped within the apolar cavity through hydrogen bonds, electrostatic interactions, and van der Waals bonds. The most commonly used materials are cyclodextrins because they have a hydrophilic exterior and a hydrophobic interior, which is suitable for application to encapsulate apolar molecules [97,105].

### 4.7. Encapsulation by Ionic Gelation

This is one of the simplest encapsulation methods in which microbeads are made from a network of biopolymers and capture the active component. The best-known gel system used is calcium alginate gel. In aqueous polymer solution, the active component is suspended and such a mixture is passed through a precision device such as a syringe, spraying or vibrating nozzle, atomizing disk, jet cutter, or others [97].

## 5. Coating Materials for Encapsulation

The coating material is of great importance in the encapsulation process, owing to its influence on target delivery and controlled release, and consequently, on the bioaccessibility of active components. It should be non-reactive with active compounds and need to have the required properties in order to protect the active compounds, such as flexibility, strength, stability, and impermeability. If the encapsulate is used in the food sector, used coating material should be “generally recognized as safe” (GRAS), inert to the encapsulating active substance, biodegradable, and strong enough to protect the encapsulated substance from the environment [106].

There are various materials used for the encapsulation of active components, such as polysaccharides from different sources (plant exudates, marine extracts, etc.), lipids, proteins, and so on [102,107,108]. The most used are gum Arabic, chitosan, and cyclodextrins [109], although, today, some more natural materials, such as whey and soy proteins, gelatin, and starch, are of more interest as coating materials because they represent the “GREEN” trend in various industries (pharmaceutical, cosmetic, and food industry) [110,111].

### 5.1. Polysaccharides

The most commonly used encapsulation materials are polysaccharides because they bind core material (active component) into an amorphous solid unit and thus allow a solid and stable capsule shape [112]. Furthermore, when the goal is to ensure the transport of bioactive substances and their release in a specific part of the digestive system, polysaccharides stand out because most of them protect the breakdown of encapsulates in the stomach and upper digestive tract, and are broken down in the colon where the most bioactive substances are absorbed [113]. Some of the frequently used polysaccharides are described below.

#### 5.1.1. Starch and Its Derivates

Starch is a polymer of α-d-glucose that consists of amylose and amylopectin and it can be modified by physical, chemical, and biochemical methods [105]. These modifications change the structure of the starch and affect the hydrogen bonds, resulting in derivatives that have a wider use compared to unmodified starch. Many components can be encapsulated with starch because it has good emulsifying properties and solubility and is inexpensive. Furthermore, it is suitable for the drying of encapsulated materials. Starch hydrolyzed derivatives also enable great protection against heat and oxidation. For the obtained derivatives, the value of Dextrose Equivalent (DE) is used, which represents the degree of starch hydrolysis. DE can have a significant effect on product properties. Higher DE decreases aqueous viscosity and a higher concentration of starch dissolves faster. On the other hand, a smaller DE allows a better structure as a wall material [97]. Recently, it has been widely used for the controlled release of encapsulated active compounds [114,115,116,117,118,119]. Maltodextrins are an example of modified starch and they are formed by partial hydrolysis of cornflour using acids or enzymes [112]. Maltodextrins are often used in extrusion [120], spray drying [121,122], fluidized bed granulation [123], and other [124,125]. Their frequent use is due to their thermal stability. In addition, maltodextrins have a neutral taste, no odor, and are easily digestible [103,120,121]. An alternative that is often used instead of maltodextrins is inulin. Inulin is a fructooligosaccharide commercially derived from chicory [112,126]. Although not much researched, inulin is suitable for use as a wall material in encapsulation because it has prebiotic properties, reduces the risk of colon cancer, enhances absorption of minerals, and many other beneficial effects [95,112,127]. Due to its viscoelastic properties, it can create soft microcapsules that are characterized by low water absorption, further leading to greater stability of encapsulated components [112,128].

Cyclodextrins are enzymatically modified starches. They are basket-shaped, hydrophobic on the inside, and hydrophilic on the outside. This structure allows them to create complexes with a wide range of organic compounds [112,129,130]. The cavity size of cyclodextrins also allows the selectivity of the components to be encapsulated [112,131]. Encapsulation of components using cyclodextrins leads to increased solubility, higher permeability through intestinal membranes, and greater bioavailability of the encapsulated compound [112,132,133].

#### 5.1.2. Cellulose and Its Derivates

Cellulose is a β-d-glucose polymer whose chains are linked by β-(1→4)-glycosidic bonds [97]. Like starch, cellulose can be modified to possess desired properties. The most commonly used cellulose derivatives are cellulose ethers, methylcellulose, hydroxypropyl methylcellulose, hydroxypropyl cellulose, and ethylcellulose. Cellulose ethers are the ones most often used for encapsulation among all cellulose derivates. Etherification improves the solubility of cellulose in organic solvents and thermoplasticity [134]. Methylcellulose and hydroxypropyl methylcellulose have similar properties and are characterized by the ability to form films and therefore are very suitable cellulose derivatives for encapsulation [135,136]. Hydroxypropyl cellulose is a cellulose derivative, soluble in water and/or ethanol. Therefore, it is suitable for encapsulation with other wall materials soluble in water, ethanol, or a mixture of both such as alginate [137,138], guar gum, gelatin, and other cellulose derivatives [109]. Ethylcellulose is a cellulose derivative apt for encapsulating components with which controlled release is desired due to drug holding capability [139,140,141].

#### 5.1.3. Plant Exudates

Plant exudates are complex mixtures of inorganic and organic molecules. They are used for various purposes in medicine, cosmetics, and the food industry and are known for their biological activities, such as antioxidant, anti-inflammatory, antimicrobial action, and many other positive effects. Depending on the species, genus, or genera, they can vary and mostly include gums, latex, resins, or root exudates and others [142].

Gums are polysaccharides of a complex structures consisting of polymers and oligomers that have a various chains and chemical structures. Leguminosae family are plant species from which the gums used in encapsulation are obtained [97].

Gum arabic (gum acacia) has a very complex structure that is still not completely clear. It contains d-galactopyranosyl units that make up the main chain to which the side chains are attached. The structure of gum arabic depends on the source from which it was obtained [112]. During encapsulation, it forms a protective film [97] and is convenient for use with other materials such as polysaccharides and derivatives, proteins, and modified starches [112,143,144,145]. Additionally, it is commonly used due to its stabilizing capacity and great emulsifying properties. By using gum arabic and other materials it is possible to improve encapsulation efficiency. For example, gum arabic around the core material creates a dried layer and prevents the contact between the core material and air, while maltodextrin creates an amorphous glass structure during encapsulation [143]. Other gums that can be used are gum Karaya, gum Tragacanth, and Mesquite gum.

Pectins are high molecular weight polysaccharides whose structure contains α-(1→4)—linked d-galacturonic acid-based units. They can be isolated from fruit peel or fruit juice residues. In terms of encapsulation, they are widely used as shell materials in double emulsion encapsulation techniques [97].

Soluble soybean polysaccharide (SSP), extracted from soybeans [97] can preserve protein particles at low pH values because acidic pH, heat, and salt do not affect solutions viscosity of SSP. It is used to stabilize emulsions, and due to its film forming and adhesion properties, it is suitable for the coating of microcapsules [146,147].

#### 5.1.4. Marine Extracts

The marine polysaccharides are very similar to cellulose and alginates and carrageenans are the most commonly used in encapsulation. Alginates are linear anionic polysaccharides composed of α-1-glucuronic acid (G) and β-d-mannuronic acid (M) linked by α-(1→4) bond. They are very abundant in nature and are produced from brown algae or the exocellular material of bacteria [94]. The functional properties of alginates depend on their structure, molecular weight, and composition. Sodium alginate is often used in encapsulation [148,149,150,151]. It can form an adaptable matrix, is non-toxic, and compatible for the encapsulation of various active components and cells. It also provides good protection for components sensitive to pH, heat, and oxygen, which is extremely important during storage [152]. Alginates react with calcium ions to form a gel structure as calcium ions bind to the carboxyl group of manuronic and galuronic acids. Carrageenans are sulfated polysaccharides isolated from red algae [97,153]. They can form gels when cations, such as K^+^ and Ca^2+^, are present [97].

#### 5.1.5. Microbial Process Polysaccharides

Xanthan, chitosan, gellan, and dextrans are polysaccharides produced by bacteria. These polysaccharides are often combined with other materials in encapsulation.

Xanthan is an anionic polyelectrolyte with high molecular weight and usually appears as calcium, potassium, or sodium salt. It is produced as an extracellular polysaccharide of *Xanthomonas bacteria*. Due to its possibility of containing cellulases, it cannot be used with cellulose derivatives during encapsulation [97,154].

Chitosan is a biodegradable polysaccharide produced by chitin partial deacetylation [97,154]. Its linear structure consists of N-acetyl-d-glucosamine and d-glucosamine linked by β-(1→4) bonds. It contains polar groups such as OH and NH_2_ that can be electron donors. Chitosan creates films and gels very well and it is frequently used [125,155]. It is soluble at low pH such as in the stomach, thus, it combines with other polymers (e.g., alginate, xanthan) to remain undigested in the stomach and is transported to the small intestine where it is broken down, after which the bioactive substance can be absorbed.

Chitosan can form hydrogels with xanthan and these hydrogels can absorb large amounts of water. The resulting chitosan-xanthan complex has a high resistance to enzymes and is suitable for encapsulation of active components in which targeted delivery and controlled release are essential [156].

Dextrans are polysaccharides with a linear polymer backbone, with mainly 1,6-α-d-glucopyranosidic linkage, which are produced by microbial fermentation of sucrose. They can be in crystalline form or hydrogel form, and have different molecular weights, all of which affect the rate of their decomposition in the GIT and the controlled release of the active substance [97,112,113].

### 5.2. Proteins

Proteins have a wide application in the encapsulation of bioactive components due to advantages such as biodegradability, biocompatibility, and good water solubility. They can be extracted from plant and animal sources. Compared to animal proteins, plant proteins are preferred because they are less allergenic [157]. Additionally, many plant proteins are apt for encapsulation of both hydrophilic and hydrophobic compounds. Proteins bind phenolic compounds by hydrogen bonds and hydrophobic interactions [104].

Isolated soy proteins are used as an encapsulation material for various purposes. They can protect sensitive components from oxidation and volatile aromatic compounds [158] or they can mask the undesirable flavors of bioactive components [147,159,160,161,162]. Soy protein isolate can be used alone [147] or in combination with other polysaccharides [163]. It is used because of various positive properties such as good gel-forming properties [164].

Gluten, which is obtained as a by-product in the isolation of starch from wheat flour, has wide application in encapsulation, either alone or in combination with other polysaccharides [97]. Viscoelasticity and low solubility in water allow easy creation of gels and films [165].

Pea proteins are another protein isolate often used in combination with polysaccharides [166,167,168]. The interactions of pea proteins and polysaccharides contribute to the formation of stable emulsions, and therefore, capsules of the desired size are formed, thus improving the encapsulation process itself [166].

Rice proteins can also be used in encapsulation [169]. The physicochemical properties of these proteins are similar to those of casein. During encapsulation, they are used with alginate or carrageenan-forming complexes [97]. Proteins extracted from rice show interesting properties that are suitable in encapsulation, such as foam and emulsion formation properties and good solubility [108].

The animal proteins used for encapsulation are caseins and gelatins. Caseins are proteins found in milk, while gelatins are obtained from collagen found, for example, in cartilage. Gelatins from fish may be highly desirable for encapsulation as they, at low temperatures, reduce water loss [97]. Gelatin is an extremely good material for spray-drying because it has the ability to form films, good solubility in water and emulsification properties, as well as the ability to create a compact structure [121].

### 5.3. Lipids

Among all lipids used as coating materials, liposomes are the most widely used [96,170,171]. Other lipids used for encapsulation processes are paraffin, waxes, and glycerides [172,173]. Liposomes are small spherical carriers. They can vary in lipid composition, size, and method of preparation. They serve to encapsulate both hydrophilic and hydrophobic components. Liposomes consist of a phospholipid bilayer that actually determines the properties of liposomes. Unsaturated phosphatidylchlorine from soybean or eggs yields unstable bilayers that are more permeable, while with saturated phospholipids containing long acyl chains, an impermeable bilayer structure is obtained [97,174].

## 6. Bioavailability of Encapsulated Phenolic Compounds

The bioavailability of phenolic compounds is affected by many diverse factors since they have to pass through several membranes and are exposed to numerous metabolic processes. Furthermore, each class or subclass of phenolic compounds has a different metabolic fate. Low water solubility and absorption, as well as rapid metabolism, lead to low bioavailability of phenolic compounds. The problem of bioavailability of phenolic compounds can be improved by controlled release in target cells. Controlled release and target delivery require the preservation of a component during its passing through the digestive system so that this same component is delivered to a place where it can be absorbed and perform its function, whether it is antimicrobial, anticancer, or otherwise. If we take this into account, encapsulation is a promising technology. The use of various coating materials in the encapsulation process can ensure greater bioaccessibility and thus the bioavailability of phenolic compounds. Regarding the bioavailability of encapsulated phenolic compounds, most of the information in the literature is about quercetin, catechins, and anthocyanins. Below some examples are discussed, and an overview of encapsulated phenolic compounds and the research conducted with them are given in the Table 1, Table 2 and Table 3.

### 6.1. Encapsulated Phenolic Acids

Very little is known about the metabolic pathway and bioavailability of encapsulated phenolic acids, and more research is needed [175]. Granata et al. [173] conducted a study where they encapsulated hydroxycinnamic acids in lipid-core nanocapsules. In vitro tests showed the stability of nanocapsules during the simulation of digestion in gastric fluids and the release of these phenolic acids into intestinal fluids [176]. In one study, ferulic acid was encapsulated in chitosan and in vivo tests were conducted. Chitosan has been shown to improve plasma retention of ferulic acid, compared with ferulic acid in free form, and thus, provide greater bioavailability [177]. Table 1 shows examples of coating materials and methods used for the encapsulation of phenolic acids.

**Table 1:** Encapsulation of phenolic acids and stilbenes.

Core Material	Wall Material	Encapsulation Method	Assay	Reference
ferulic acid	chitosan-tripolyphosphate pentasodium	ionic gelation	in vitro cytotoxicity	[177]
	poly-D,L-lactide-co-glycolide (PLGA)	double emulsion	in vitro release, in vitro anti-tumoral activity	[178]
caffeic acid	poly-D,L-lactide-co-glycolide (PLGA)	emulsion	in vitro release	[179]
syringic acid	D-Alpha tocopheryl polyethylene glycol 1000 succinate (TPGS)	thin-film dispersion	pharmacokinetic studies, tissue distribution, in vivo antioxidant activity	[180]
trans-resveratrol	zein	electrospraying	in vitro release study, in vitro digestion, intestinal permeability	[181]
	poly-D,L-lactide-co-glycolide (PLGA)	precipitation	in vitro release, in vivo biodistribution, in situ single-pass intestinal perfusion	[182]

### 6.2. Encapsulated Flavonoids

Table 2 gives an overview of flavonoid encapsulation using different encapsulation methods and coating materials.

**Table 2:** Encapsulation of flavonoids.

Core Material		Wall Material	Encapsulation Method	Assay	Reference
flavanols	quercetin	chitosan	ionic gelation	in vitro release and cytotoxicity, in vivo anti-tumor activity and biodistribution	[183]
		poly(lactic-co-glycolic acid) (PLGA)	emulsion diffusion evaporation	in vitro release	[184]
		soluplus micelles	film dispersion	in vitro release, in vivo pharmacokinetics	[185]
		linseed oil, GMS, P6, Tween 80, 1,1-propylene glycol	high pressure homogenization	in vitro release	[186]
		poly-D,L-lactide (PLA)	solvent evaporation	in vitro release	[187,188]
		glycerol monostearate (GMS), medium chaintriglycerides (MCT), soy lecithin	emulsifying and solidifying	in vitro release, in vivo tissue distribution	[189]
		zein, 2-hydroxypropyl-β-cyclodextrin	spray-drying	in vitro release	[190]
		casein, 2-hydroxypropyl-β-cyclodextrin	coacervation	in vitro release	[191]
		poly(lactic-co-glycolic acid) (PLGA)	solvent displacement	in vitro release	[192]
		ethylcellulose	precipitation	in vitro release, ex vivo skin penetration	[193]
		soy lecithin, glyceryl tridecanoate, glyceryl tripalmitate, vitamin E acetate, Kolliphor HS15	phase inversion	in vitro release, cytotoxicity, cellular uptake, and apoptosis assay in breast cancer cells	[194]
		(β-CD)-dodecylcarbonate	freeze-drying	cell viability	[195]
	kaempferol	chitosan, sodium tripolyphosphate	ionic gelation	in vitro release	[196]
		lecithin–chitosan	electrostatic self-assembly	in vitro release, antifungal activity	[197]
	fisetin	DOPC, cholesterol, DODA-PEG2000	liposomes	in vivo	[198]
		PLGA (poly-lactide-co-glycolic acid), HPβCD (hydroxyl propyl beta cyclodextrin)	emulsion, freeze drying	in vitro release, in vivo studies	[199]
flavones	tangeretin	zein	emulsion	in vitro digestion	[200]
	apigenin	soybean oil, Tween 80		in vitro digestion, in vivo pharmacokinetics	[201]
	rutin	chitosan	ionic gelation	simulated gastrointestinal digestion	[202]
flavanones	naringenin	phospholipid, cholesterol, sodium cholate, and isopropyl myristate	liposomes by thin-film dispersion	in vitro release, in vivo studies	[203]
flavan-3-ols	epigallocatechin gallate (EGCG)	gum arabic, maltodextrin	spray drying	retention/release studies	[204]
		chitosan-tripolyphosphate	freeze-drying	stability in gastrointestinal tract of the mice	[205]
	catechin hydrate	phosphatidylcholine (PC)	liposomes	in vitro release, ex vivo permeation, in vivo pharmacokinetics	[206]
		horse chestnut, water chestnut and lotus stem starch	freeze drying	simulated gastrointestinal digestion and bioactivity retention, antidiabetic properties	[207]
	green tea catechins	soy protein	emulsion	in vitro digestion, epithelial permeability	[208]
		vitamin C and xylitol, γ-cyclodextrin and hydroxypropylmethyl cellulose phthalate	film-forming	in vitro digestion, transport study	[209]
		hydroxypropyl methyl cellulose phthalate	coating	in vitro digestion, transport study	[210]
	tea catechins	corn oil and polysorbate 80	emulsion	in vitro simulated digestion	[211]
isoflavones	daidzein	phospholipid	film-homogenization	distribution in the GI tract, in situ intestinal absorption, in vitro release	[212]
	genistein	Soluplus^®^ and Vitamin E d-α-Tocopheryl polyethylene glycol 1000 succinate (TPGS)	organic solvent evaporation	in vitro release, transport study, in vivo pharmacokinetics	[213]

The most common flavonoid that can be found in fruits and vegetables in large quantities is the flavonol quercetin. It is characterized by high antioxidant activity and plays a major role when it comes to diseases of the colon [214]. As it is found in plants in the form of water-soluble glycosides, it is rapidly hydrolyzed in the small intestine. The desirable characteristics of quercetin are limited due to its low water solubility in the physiological medium, and it is not stable. For these reasons, its biological activity is limited. Encapsulation in biodegradable polymers can improve the permeability and stability of quercetin and thus its bioavailability [187]. The goal of quercetin encapsulation is to preserve it as it passes through the stomach and small intestine and to allow its release in the large intestine [215]. Singhal et al. [216] conducted an in vitro study where guar gum was shown to be a desirable material for encapsulation of quercetin due to its ability to preserve the active compound during its passage through the upper part of the GIT. Guar gum is profoundly branched galactomannans and due to that structure, it holds out against enzymatic breakdown in GIT [216]. The combination of chitosan and xanthan gum can be promising for the controlled release of quercetin. Microparticles prepared in this way enabled resistance in an acidic environment and a complete release in alkaline conditions [214].

The flavonoid fisetin has low water solubility and, similar to many other phenolic compounds, has antitumor activity. By applying nanoemulsion encapsulation and administering such encapsulated fisetin intraperitoneally, the bioavailability of fisetin was increased 24-fold. Fisetin bioavailability is thought to be increased due to faster absorption of fisetin nanoemulsion from the peritoneal cavity compared to free fisetin [217].

Many studies have shown that anthocyanins have a positive effect on changing the microbiota of the colon, which explains their great contribution to human health [87,218,219].

When talking about catechins (monomeric flavanols) such as catechin, epicatechin, epicatechin gallate, EGC, and EGCG are most often mentioned. Green tea is very rich in these compounds, and research has mostly focused on the extraction of catechins from green tea [220]. In vitro studies have shown that low concentrations of catechins are sufficient to have a positive effect on health, but levels of catechins in the bloodstream after oral administration are usually extremely low [221]. Due to poor stability in the GIT and limited permeability and transport across intestinal membranes, their positive effect is limited [222,223,224]. Protecting catechins from environmental conditions found in the small intestine can improve the desired effects on human health. Green tea catechins encapsulated with soy protein nanoemulsion have been shown to increase bioavailability compared to non-encapsulated ones [208]. The most commonly-used material for EGCG encapsulation is chitosan. Encapsulation of EGCG in the chitosan-tripolyphosphate nanoparticles showed an increase in the amount of EGCG in the bloodstream. Absorption is thought to increase because chitosan-tripolyphosphate combination is more resistant to conditions in the small intestine [205]. Additionally, higher concentrations of catechin metabolites have been reported in a study conducted with wine enriched with phenolic compounds in the form of nanoparticles encapsulated by a combination of zein and L-lysine [225]. EGCG can also be encapsulated in materials such as modified β-lactoglobulin, where slower degradation can be seen compared to free EGCG [226]. Nanoemulsion system oil in water is demonstrated as a way to increase EGCG absorption along with controlled release [211].

Cyclodextrins are another material used in the catechins encapsulation. Son et al. [209] used γ-cyclodextrin and hydroxypropylmethylcellulose phthalate and obtained results that showed the stability of encapsulated catechins from green tea during digestion. In addition, better intestinal transport of catechins coated with hydroxypropylmethylcellulose phthalate has been shown. These results are also visible from a previously conducted study where the bioavailability of catechins from green tea increased coating in hydroxypropylmethylcellulose phthalate [210]. Hydroxypropylmethylcellulose phthalate does not dissolve at the acidic conditions present in the stomach but dissolves under the alkaline environment of the small intestine. In a recent study, catechin loaded starch-based nanoparticles were produced. Used starch was from different sources: water and horse chestnut and lotus stem. An in vitro study showed sustained bioactive properties of catechins [207].

Anthocyanins are a subclass of flavonoids, but due to their numerous positive biological effects (prevention of diabetes and colon cancer, antioxidant activity, and anti-inflammatory properties) [227,228], they are often studied as a separate group. Nevertheless, as mentioned earlier, almost all anthocyanins ingested through the diet are degraded in the small intestine due to their sensitivity to the present conditions. By changing the pH to alkaline, anthocyanins lose protons and are being translated into an unstable form, the quinoidal base [229], resulting in low bioavailability.

Various encapsulation techniques have been proposed as a solution to improve the low bioavailability of anthocyanins [230]. Pectin, sodium alginate, and maltodextrin have been proven to be beneficial for anthocyanins encapsulation (Table 3) [105,231,232], while cyclodextrins were singled out. Cyclodextrins have a torus-shaped structure and hydrophobic cavity that is adaptable and due to the amphiphilic character of anthocyanins. Therefore, they are excellent wall material for anthocyanins encapsulation [233]. According to Flores et al. [233], the time required for 50% of malvidin-3-glucoside to be metabolized was 3 h, while in a study conducted by Hidalgo et al. [87], 1 h was required. These results showed that it is possible to achieve a controlled release of anthocyanins in the colon [87,233]. Comparing bilberry extract encapsulated by whey protein and citrus pectin, it is noted that the coating formed utilizing of whey protein allows short-term bioavailability. On the other hand, citrus pectin allows the availability of billberry extract anthocyanins during passage through the small intestine resulting in the formation of phloroglucinol aldehyde in plasma [234]. Ferritin has been shown to be a thankful protein coat for anthocyanins encapsulation. Using the Caco-2 cell model, cyanidin-3-*O*-glucoside in apoferritin nanocapsules was shown to be more efficiently transported compared to the free one [235]. Zein protein nanocapsules of wine extract improved the bioavailability of malvidin-3-*O*-glucoside to some extent [225]. Anthocyanin encapsulation can also be performed using a combination of multiple materials and using double-walls forms. Ge et al. [236] used a combination of chitosan hydrochloride and carboxymethyl chitosan capsules and double-walls form of encapsulation using chitosan and β-lactoglobulin. In both cases, anthocyanins increase in the stability and bioavailability was demonstrated using in vitro digestion methods [236].

**Table 3:** Encapsulation of anthocyanins.

Core Material ^1^	Wall Material	Encapsulation Method	Assay	Reference
blackberry purees	β-cyclodextrin	molecular inclusion	in vitro digestion	[237]
saffron anthocyanins	β-glucan and β-cyclodextrin	spray drying	in vitro digestion	[238]
*Vaccinium ashei* extracts	whey protein isolate	spray drying	in vitro digestion	[239]
*Bryophyllum pinnatum* extract	β-cyclodextrin	emulsion	in vitro antioxidant activity, anti-inflammatory activity	[240]
bran extract	maltodextrin, gum arabic, whey protein isolate	spray drying	in vitro digestion	[241]
	alginate-whey protein isolate	ionic gelation		
sour cherries skins extract	whey proteins isolate	freeze-drying	in vitro digestion	[100]
bilberry extract	whey protein, citrus pectin	emulsification and thermal gelation	human pilot study	[234]
anthocyanins standards mixture	cyclodextrins	freeze-drying	in vitro fermentation	[233]
anthocyanins standards mixture	chitosan hydrochloride, carboxymethyl chitosan, β-Lactoglobulin	ionic gelation	in vitro digestion	[236]
bilberry extract	pectin amide	extrusion	in vitro digestion	[242]
	pectin amide with an additional shellac coating	emulsification/heat gelation		
	whey proteins	spray drying		
black carrot extract	polycaprolactone	double emulsion	in vitro release and cytotoxicity	[243]
	cholesterol and non-ionic surfactant (Tween 20)	niosome method		
mulberry-extracted anthocyanin	alginate/chitosan	spray drying and external gelation	in vitro degradation and release	[244]
red pepper waste	whey protein	spray drying and freeze-drying	in vitro digestion	[101]
bilberry extract	whey protein isolate	gelation	in vitro digestion	[245]

^1^ Source of anthocyanins.

Chitin is also a good choice as wall material for anthocyanins encapsulation. The porous structure, hydrogen bonds, and hydrophobic interactions of chitin contribute to better anthocyanin binding. Using an additional layer of ethyl cellulose with chitin increased the possibility of anthocyanins passing through the small intestine to the colon [246]. Anthocyanin nanoparticles encapsulated in chitosan, chitin derivative degraded more slowly under simulated digestion conditions than those found in free form in solution [227]. A combination of chitosan combined and alginate in the encapsulation mulberry extract rich with anthocyanins was found to be the most suitable due to the high encapsulation efficiency and resistance under digestive simulation conditions. Chitosan, which is positively charged, reduced the negative charge of the capsules due to electrostatic interactions during the intrusion of alginate beads loaded with anthocyanins into a calcium chloride solution containing 0.05% chitosan [244]. By comparing encapsulation methods, liposomal powders with chitosan provided better protection of anthocyanins under conditions of increased pH and temperature compared to the spray drying method [247].

Innovative encapsulation technology of vesicular drug delivery systems, niosome, can significantly improve target delivery, as it allows direct delivery of the drug to the part where it is needed. This reduces the amount needed to administer in order to achieve the desired impact [248]. Niosome preparation and double emulsion were used to encapsulate anthocyanin black carrot extract. Capsules were prepared with polycaprolactone as a coating material. According to in vitro data, just about 90% of anthocyanins were released within 10 h making this new niosomal technique suitable for anthocyanin delivery [243].

### 6.3. Other Encapsulated Phenolic Compounds

In vitro studies and studies on rats are preformed using nanoparticles of stilbene resveratrol encapsulated in casein. In vitro studies showed that the nanoparticles were stable in pH conditions of simulated gastric and intestinal fluids. Additionally, the same research was conducted with rats. Orally administered encapsulated resveratrol was able to reach the intestinal epithelium and plasma resveratrol concentrations were high and constant in the plasma. The results showed that the oral bioavailability of resveratrol encapsulated in casein was 10 times higher than when the resveratrol solution was administered [249]. Resveratrol encapsulated as a stearic acid-based solid lipid nanoparticle with poloxamer 188 used for coating of capsules may also serve to improve bioavailability with the delayed release [250]. Other examples of encapsulation of trans-resveratrol are given in Table 1.

Curcumin is another phenolic compound whose low water solubility results in low bioavailability. Li et al. [251] encapsulated curcumin in β-lactoglobulin and investigated permeability using a Caco-2 cell model. The results showed that the encapsulation of curcumin using β-lactoglobulin improves the solubility and stability of curcumin under pH conditions during simulated GI digestion. In this way, the resistance to the enzyme pepsin was increased, but also the sensitivity to trypsin. Using the ionotropic gelation encapsulation method by combining a lipid and polymer delivery system, curcumin was encapsulated. This encapsulation method has been shown to be desirable for the delayed release of phenols that have low solubility, are lipophilic, and have low oral bioavailability [252].

## 7. Future Prospective

It is indisputable that phenolic compounds from plants play a significant role in the prevention of many diseases in the human body, as numerous studies show. However, in order to use all their potentials, it is necessary to ensure their absorption, i.e., it is crucial to know and understand their bioaccessibility and bioavailability. Although in recent years the focus of much research is in this field, there are still many unknowns. In vitro simulated digestion tests are most frequently used in an effort to explain the absorption of phenolic compounds in the gastrointestinal tract. In terms of phenolic compounds bioavailability, encapsulation is a promising technique. It ensures the controlled supply and release of phenolic compounds to the target site in the test system where the best absorption of the active substance and its transfer to the tissues can be enabled. The effectiveness of the controlled delivery and release of the active substance, in this case, phenolic compounds, depends primarily on their structure, their interactions with other food ingredients, and among themselves, the properties of the encapsulation material and the encapsulation method used, but also on the intestinal microflora. Because phenolic compounds are structurally diverse, future research should focus on the development of encapsulation methods and new encapsulation materials to improve their bioavailability. The reason why encapsulation is not wider applied is the problem of scale-up and low productivity.

The most in vitro models for testing the absorption of active substances in the gastrointestinal tract are based on two-dimensional cell cultures. A new technique, organ-on-a-chip systems roughly capture the three-dimensional (3D) structure of living cells. Such cell systems can recap the necessary organ functions and thus allow better examination of these functions in vitro. The great advantage of this system is that various components can be constantly tested and these systems very well illustrate the environment such as in vivo [253]. In recent years, more and more research has focused on the development of rapid tests that recreate digestive conditions. In addition to the organ-on-a-chip technique, further research can be directed to a similar method using microchannels. Microchannels or microreactors are used in fields of biochemistry and chemistry and have a number of advantages such as greater process control and safety, better scale-up capabilities and higher process selectivity. In addition, the use of microreactors reduces the consumption of time and chemicals [254].

The trend in research in recent years has focused on the utilization of agro-food industry residues/waste in the production of high-value products such as enzymes, extracts rich in phenolic compounds, as well as the production of food enriched with production residues or extracts obtained from them.

Solid-state fermentation (SSF) is an environmentally friendly and cost-effective method of exploitation of highly abundant lignocellulosic materials as substrates for fungi cultivation. It has been proven that the treatment of materials rich with phenolic compounds by fungi during SSF increases the bioaccessibility of phenolic compounds in the digestive system [255].

Additionally, some of the enzymes that could be used in vitro simulated digestion methods, e.g., lipase [256], can be produced by the use of fungal-based SSF.

Finally, lignocellulosic materials could be an affordable source of encapsulation material due to their chemical composition, which would contribute to the development of a cost-effective encapsulation method. One example is pectin, which is found in plants and food industry residues (fruit pomace and peel). After isolation, it can be used as a gelling agent in encapsulation [257]. Another example is the use of waste from the orange juice industry. By producing a dietary fiber-rich powder, a new coating material can be obtained to replace maltodextrins for the spray-drying method [258].
